# Retraction Note: p53 destabilizing protein skews asymmetric division and enhances NOTCH activation to direct self-renewal of TICs

**DOI:** 10.1038/s41467-026-77491-3

**Published:** 2026-09-14

**Authors:** Hye Yeon Choi, Hifzur R. Siddique, Mengmei Zheng, Yi Kou, Da-Wei Yeh, Tatsuya Machida, Chia-Lin Chen, Dinesh Babu Uthaya Kumar, Vasu Punj, Peleg Winer, Alejandro Pita, Linda Sher, Stanley M. Tahara, Ratna B. Ray, Chengyu Liang, Lin Chen, Hidekazu Tsukamoto, Keigo Machida

**Affiliations:** 1 Department of Molecular Microbiology and Immunology, University of Southern California, Los Angeles, CA 90033 USA; 2 Department of Chemistry and Biological Sciences, University of Southern California, Los Angeles, CA 90089 USA; 3 Department of Medicine, University of Southern California, Los Angeles, CA 90033 USA; 4 Department of Surgery, University of Southern California, Los Angeles, CA 90033 USA; 5 Saint Louis University, St. Louis, MO 63103 USA; 6 Department of Pathology, Keck School of Medicine, University of Southern California, Los Angeles, CA 90033 USA; 7 Southern California Research Center for ALPD and Cirrhosis, Los Angeles, CA 90033 USA; 8 VA Greater Los Angeles Healthcare System, Los Angeles, 90073 CA USA; 9 Present Address: Section of Genetics, Department of Zoology, Aligarh Muslim University, Aligarh, 202002 India; 10 Present Address: Department of Genetics and Genomics, and The Jackson Laboratory for Genomic Medicine, University of Connecticut Health, Farmington, 06032 CT USA

Retraction to: *Nature Communications* https://doi.org/10.1038/s41467-020-16616-8, published online 17 June 2020

The Editors have retracted this article. After publication, concerns were raised regarding image similarities within the article and with other publications from shared authors. Specifically:

- Fig. 1D, Ethanol WD and Fig. 1E, Ethanol WD in^1^ appear to be similar
- Fig. 1D, Tbc1d15^FL/FL^; NS5A and Fig. 2B, Core in^1^ appear to be similar
- Fig. 2E, LGN and Fig. 3G, N1ICD appear to be similar (flipped horizontally)
- Fig. 5B, β-actin and Fig. 1a, β-actin in^2^ appear to be similar
- Fig. 5E, aPKCζ and Fig. 6I, Actin in^3^ appear to be similar
- Fig. 5E, aPKCζ (IP: alpha-AURKA) and Fig. 2D, SETd7 and Fig. 2L, TLR4 in^4^ and Fig. S1B, PTEN in^5^ appear to be similar
- Fig. 6E, β -actin and Fig. 3G, β-actin (sh-EED) in^6^ appear to be similar

The authors stated that the errors occurred due to inadvertent incorporation of the images. However, upon request, the authors couldn’t provide appropriate original data nor a satisfactory response to the concerns raised. Due to this, the Editors no longer has confidence in the presented data.

Author Keigo Machida has stated that he and the following authors agree to retraction; Hye Yeon Cho, Hifzur R. Siddique, Mengmei Zheng, Yi Kou, Da-Wei Yeh, Tatsuya Machida, Chia-Lin Chen, Dinesh Babu Uthaya Kumar, Linda Sher, Stanley M Tahara, Ratna Ray, Chengyu Liang, Lin Chen, Hidekazu Tsukamoto. Authors Vasu Punj, Peleg Winer, Alejandro Pita could not be contacted by the publisher.
